# The evolution of cheating in viruses

**DOI:** 10.1038/s41467-021-27293-6

**Published:** 2021-11-26

**Authors:** Asher Leeks, Stuart A. West, Melanie Ghoul

**Affiliations:** Department of Zoology, University of Oxford, Oxford, OX1 3PS UK

**Keywords:** Social evolution, Virology

## Abstract

The success of many viruses depends upon cooperative interactions between viral genomes. However, whenever cooperation occurs, there is the potential for ‘cheats’ to exploit that cooperation. We suggest that: (1) the biology of viruses makes viral cooperation particularly susceptible to cheating; (2) cheats are common across a wide range of viruses, including viral entities that are already well studied, such as defective interfering genomes, and satellite viruses. Consequently, the evolutionary theory of cheating could help us understand and manipulate viral dynamics, while viruses also offer new opportunities to study the evolution of cheating.

## Abstract (web summary)

Cooperation is vulnerable to cheating, and both cooperation and cheating may be especially common in viruses. Here, Leeks et al. place diverse examples of viral cooperation and cheating within an evolutionary framework, highlighting opportunities emerging from greater synthesis of virology and evolutionary theory.

## Introduction

Cooperation can be observed at all scales of biology. Bacteria secrete molecules that scavenge resources for the local group of cells, worker ants forage for food to rear the offspring produced by their queen, and subordinate meerkats babysit the offspring of the dominant individuals in their group^1–3^. We term these kinds of acts or traits cooperative because their evolution is influenced by the benefit they provide to other individuals, not just to the individual that performs the act^4^.

Whenever cooperation occurs, there is the potential for exploitation by ‘cheats’, that avoid the cost of cooperating, but still gain the benefits of others cooperating^5,6^. For example, bacterial mutants that have lost the ability to produce and secrete a molecule that benefits the local group, but can still benefit from the molecule secreted by neighbouring cells^7–9^. However, the extent to which cheating occurs in nature has proved contentious, and empirical examples of cheats are relatively rare^6,10^. The prevalence of cheating matters, because it determines the extent to which different individuals are in conflict over cooperation, and whether successful cooperation needs mechanisms to counter cheating^5,6,11,12^.

In this perspective, we suggest that, in contrast to elsewhere in the living world, cheats are both common and relatively easy to detect in viruses. Several examples of viral cheats are already well studied within virology, including defective interfering genomes and satellite viruses. We synthesise the relevant evolutionary and virology literatures, showing how similar issues have been examined in these two fields, but from very different perspectives. We suggest that the widespread prevalence of cheating in viruses poses novel evolutionary questions, the answers to which will have direct implications for our ability to manage viral infections.

We start by defining cooperation in the evolutionary sense, noting that the evolutionary definition of cooperation differs from how the term is often used in virology. We then define cheating, discuss how both cooperation and cheating can occur in viruses, and show how to test experimentally whether a viral entity is a cheat. Next, we survey the main types of viral cheats, suggest whether further viral cheats may be found, and consider how social evolution theory can help us to understand the distribution and dynamics of viral cheats. Finally, we discuss how the study of viral cheating can challenge and expand evolutionary theories of cheating, while also offering virologists new ways to understand and manipulate viral population dynamics.

## What is cooperation?

Before discussing viruses, it is useful to define exactly what we mean by both cooperation and cheating. When an individual is cooperating, it is performing an act or trait that is maintained by natural selection because it benefits another individual^4^. Cooperation poses an evolutionary problem because, all else being equal, it should reduce the relative fitness of the individual performing the cooperation, and hence be selected against (Box 1). The evolutionary definition of cooperation that we use here differs from the way that cooperation is often used in virology, because our definition excludes viral traits that benefit other individuals as a by-product, but that are maintained by natural selection solely due to benefits to the trait-holder.

There are two broad solutions to this problem of cooperation^1^. Firstly, cooperation will be favoured if it provides a net benefit to the individual performing the cooperation. This may occur if cooperation yields return to the cooperator, such as reciprocation by the recipients of cooperation, or if failure to cooperate entails costs, such as sanctioning by partners. A variety of such mechanisms have been described that can result in initially costly cooperation being compensated in the long run^1^.

The second solution to this problem is that cooperation can be favoured if it provides a benefit to other individuals that carry the cooperative gene, even if it is costly overall to the cooperative individual. This process is termed kin selection, because the easiest and most common way for individuals to carry the same genes is through common descent. By helping a close relative reproduce, an individual is still passing on its genes to the next generation, just indirectly^13^. Kin selection (indirect benefits) has been shown to explain many forms of cooperation, from the production of shared molecules in bacteria, to the evolution of sterile workers in the social insects^14^.

Box  1  Evolutionary  definitions  for  cooperation  and  cheating We use the evolutionary definitions for both cooperation and cheating. We define cooperation as a trait that increases the lifetime reproductive success of another individual, and has evolved at least partly because of this benefit. We define cheating as: (i) a trait that is beneficial to a cheat and costly to a cooperator in terms of inclusive fitness; (ii) when these benefits and costs arise from the cheat benefitting from cooperation, rather than another cooperator^5^.

Our definition of cheating is relatively broad, encompassing all cases when cooperation is exploited. An alternative, narrower definition requires that a cheat also has to have evolved from a cooperative lineage^6^. According to this narrower definition, conspecific avian brood parasites would be cheats, but brood parasites that are different species, such as cuckoos, would not. When applied to viruses, this narrower definition would still classify a range of cases as cheating, such as defective interfering genomes, and point-mutation mutants such as D51 and PhiH2. However, according to the narrower definition, other cases would still be parasites, but would no longer be counted as cheats; these include satellite viruses, since these have unclear origins and may not have evolved from the cooperator they exploit, and therapeutic interfering particles, since these are manufactured rather than naturally evolved. The narrower definition would also consider cases where a defective interfering genome exploits a different type of virus from which it evolved to not be a cheat. The broader definition that we use counts all of these as cheats, but then classifies them into different types of cheat (Figs. 4 and 5)^5^.

The broader framework that we use emphasises functional similarities, in terms of fitness consequences, between entities that may otherwise appear quite different. For example, we can draw links between defective interfering genomes, that arise through mutation, and satellite viruses, that coexist over long timescales; or between defective interfering genomes, that arise naturally, and therapeutic interfering particles, that are synthesised artificially (Figs. 4 and 5). In addition, we provide an approach for further classifying different types of cheat, such as those that spread within but not between hosts (short-sighted) or those that spread both within and between hosts (long-sighted) (Figs. 4 and 5).

By our definition, cheating is context-dependent, meaning that an individual that produces less of something can be a relative cheat, compared to an individual that produces more^50^. Consequently, a spectrum of cheating is possible, between ‘full cheats’ that completely lose the ability to cooperate, and ‘partial cheats’ that keep some or most ability to cooperate
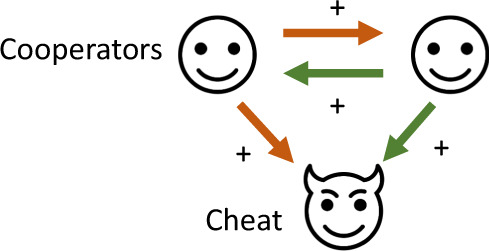

## What is a cheat?

Cheats are individuals that exploit cooperators, by avoiding paying the cost of cooperation, while still benefiting from the cooperation of others^5,6^ (Box 1). Cheating can be thought of as a special form of parasitism, in which the parasite exploits a social trait, benefitting from the trait in the same way that the trait has evolved to be used by cooperators. Cheating, therefore, exerts specific selection pressures that can select against cooperation, and/or shape the evolution of cooperative traits. This can occur even if the cooperative trait is essential for survival, potentially leading to population extinction if cheats reach a high enough frequency. While this paper focuses on the cheating perspective, which has only rarely been used within virology, some of the viral entities we discuss have also been modelled as parasites in the past, and both perspectives can be helpful^15,16^.

**Fig. 1:**
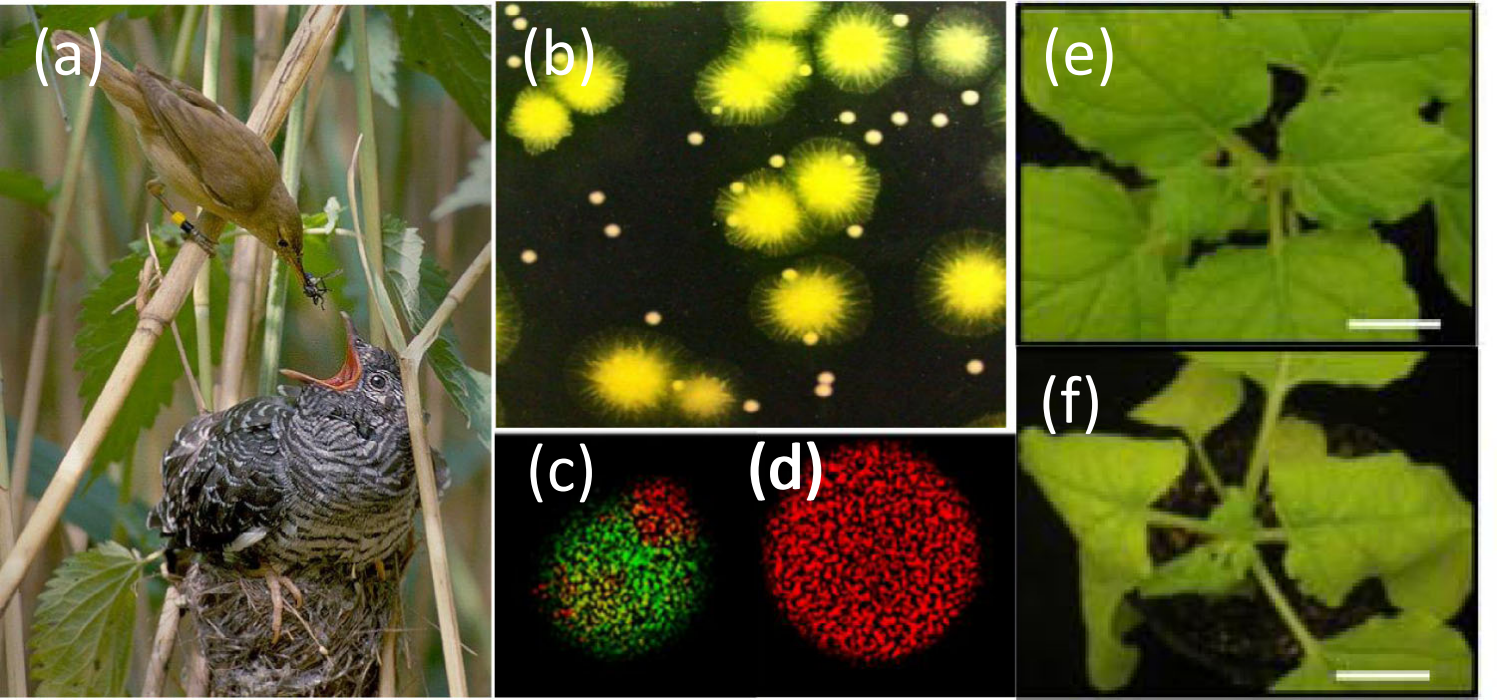
Cheating occurs throughout the natural world, including in viruses. **a** The common cuckoo (*Cuculus canorus*) lays eggs in other birds’ nests, here tricking a reed warbler (*Acrocephalus scirpaceus*) into taking care of a much larger cuckoo chick^21,143^. **b** Cells of the bacterial pathogen *Pseudomonas aeruginosa* which do not produce iron-scavenging molecules (labelled in green) are able to exploit those produced by others (labelled in white), and consequently grow much larger colonies^5^. **c**, **d** In Vesicular Stomatitis Virus (VSV), when a defective interfering genome (labelled in green) is grown in a mixed infection with wild-type VSV (labelled in red), the defective interfering genome exploits replicase proteins encoded by the wild-type cooperator, resulting in a colony (**c**) that is dominated by the defective interfering genome, and grows less effectively than a colony consisting just of the cooperative wild-type (**d**)^144^. **e**, **f** In cucumber mosaic virus (CMV) infections, a satellite (satCMV) exploits gene products encoded by the wild-type, substantially reducing the overall viral load and leading to less severe infections in plants infected by both satellite and wild type (**e**) compared to plants infected by just the wild type (**f**)^145^. Panel **a** from Per Harald Olsen (CC BY-SA 3.0), no changes made, reference ^146^. Panel **b** from Melanie Ghoul, no changes made, reference ^5^. (c and d) from John Yin, no changes made, ref. ^143^. **e**, **f** from Zhiyou Du, no changes made, ref. ^144^.

The simplest possible form of cheating is to just not cooperate. Bacteria have provided numerous examples of individuals that cheat by ‘not cooperating’. For example, when the iron is limited, bacteria produce and release siderophores, which are molecules that scavenge iron from the environment and make it available to the bacteria. Siderophores provide a benefit to the local group of cells, by allowing all local cells to access iron, not just the cell that produced the siderophores. Consequently, siderophores represent a form of cooperation that is termed a ‘public good’ (Box 2)^8,17,18^. Cells that do not produce siderophores are still able to take up iron via siderophores produced by other cells, and so represent a form of cheat. Cheats that exploit siderophores, and other ‘non-producing’ cheats that exploit similar bacterial public goods, have been observed in both laboratory and natural populations of bacteria (Fig. 1)^7,19,20^.

Cheating can also take more active or devious forms, such as in avian brood parasitism (Fig. 1). Here, females add their eggs to another’s nest, where the offspring of the cheat is then raised by the host parents, to the detriment of the host’s own offspring. Avian brood parasites have evolved a range of adaptations in order to exploit the host, including eggs that mimic those of the host, begging behaviour that allows their chicks to out-compete the host’s own chicks for parental resources, and sometimes even ejection of the host’s offspring from the nest^21^. There are parallels here with the adaptations that some viral cheats have evolved in order to exploit cooperative viruses, which can include structural changes that allow the cheat to out-compete the cooperator, or the addition of genetic sequences that allow the cheat to make more efficient use of viral public goods^22–25^.

Box 2  Cooperation  as  a  public  goods  game Many forms of cooperation in microorganisms such as bacteria and viruses are analogous to what economists and evolutionary biologists call a public goods game. In the simplest public goods game, there are *N* unrelated group members who can each contribute some resources to a group project. Those resources are then multiplied by a factor *M*, and divided out amongst each member of the group, such that each individual gains *M/N* per unit of resources contributed.

This game illustrates the problem of cooperation (Box 1). Cooperation by producing public goods is favoured at the group level—if all individuals cooperate, everyone does better. However, if each individual gains back less than one unit for each unit that they invested (when *M*/*N* < 1), then each individual does better if they invest nothing (not cooperate). Selfish interests are increasingly likely to outweigh the benefits of cooperation as *N* increases, since higher *N* means that the benefits of cooperation have to be shared with increasing numbers of other individuals. Even though each individual gets a return on their own investment, they can still be selected to invest nothing.

Public goods games do not require individuals to make decisions. All that is required is that individuals can evolve to invest different amounts of resources into the public good. Then, natural selection will favour the individuals that invest the optimal amount of resources for increasing their fitness, ultimately leading to well-adapted organisms that appear to ‘play’ the game correctly.

Viruses can play public goods games whenever there are multiple viral genomes in a cell (*N* > 1). We expect multiple genomes to commonly be present, since even if a cell is initially infected by just one viral genome, that genome will be replicated, quickly resulting in large numbers of viral genomes^151^. Consequently, the production of shareable gene products can be seen as a public goods game that is open to cheating (*N* > 1). Viral gene products that can be shared between multiple genomes, and therefore act as public goods, are common and are known as ‘trans-complementable’ or ‘trans-acting’ within virology. These public goods in viruses are closely analogous to those in bacteria and other microorganisms, which produce shared gene products such as elastase, iron-scavenging siderophore molecules, or beta-lactamase antibiotics^17^. In viruses, many essential viral gene products can be shared, and therefore act as trans-complementable public goods. For example, the universally required replicase enzymes, which replicate the viral genome, and capsid proteins, which construct the capsid shell that encloses the viral genome. Public goods games are therefore crucial in viruses, since almost all viruses depend on some kind of public good to complete their lifecycle^30^.
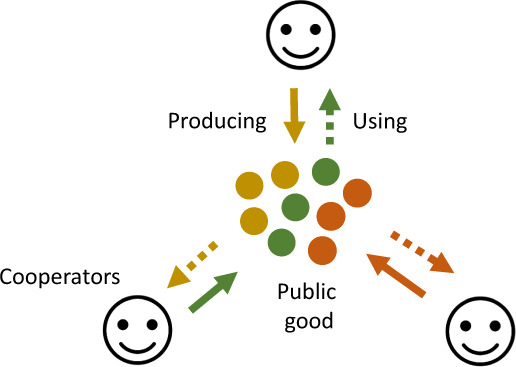

## How do viruses cooperate?

When examining cooperation, we need to think about interactions between different ‘individuals’. In viruses, we consider each physically distinct copy of a viral genome to be an individual, because it is the largest unit that we can consider to be acting as a single agent. Larger groupings such as a virion or cloud of genome sequences can contain multiple distinct genetic entities that can be in evolutionary conflict, and so cannot be considered an individual^26–29^.

**Fig. 2:**
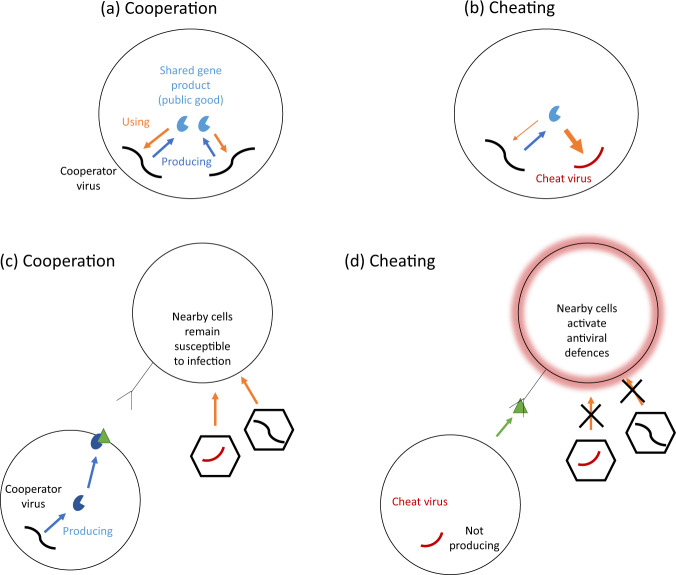
Viruses can cooperate when infecting the same cell, and also when infecting different cells. **a** In coinfection, shared viral gene products, such as replicase enzymes or capsid proteins, have the potential to benefit other viral genomes, and hence act as cooperative ‘public goods’ (Box 2). **b** When multiple viral genomes infect a host cell, there is also the potential for cheating, where some individuals benefit from the public good without producing it (cheats). **c** Cooperation can also occur between viral genomes that infect different host cells. Here, cooperative viruses produce a gene product that prevents host cells releasing interferon, keeping the local population of host cells susceptible to infection, and hence providing a benefit to other viral genomes infecting different cells^5^. **d** Cheat viruses do not block interferon, and hence replicate faster than cooperators. This cheating is costly for the viral population as a whole, because interferon is released from the infected cell, binding to nearby cells, which become resistant to infection by other viral genomes.

The simplest and most common form of cooperation in viruses can occur when multiple viral genomes infect the same host cell and share gene products (Box 2). For example, when one genome produces a replicase enzyme, this will commonly replicate all the genomes in the host cell, and not just the genome that produced it (Fig. 2). In this case, the shared replicase enzyme provides both a direct benefit to the genome that produced it, and a shared benefit to other genomes in the cell. When gene products such as replicase enzymes are shared between genomes, they are potentially cooperative, and termed ‘public goods’ by evolutionary biologists, or ‘trans-acting’ by virologists (Box 2).

Other viral gene products can also act as public goods, provided they are shared between multiple viral genomes (Box 2 and Fig. 2). For example, capsid proteins build the viral capsid (or virion) that transports viral progeny to new cells, and sometimes viral capsids can contain genomes other than those that produced the capsids. The production of shared replicase enzymes and capsid proteins together represent two potentially very common forms of cooperation in viruses. More generally, cooperation is important for the evolution of any shared viral gene product that has evolved at least partially because of a group benefit that it provides^30^.

The extent to which gene products such as replicase and capsid proteins act as cooperative public goods depends on the extent to which they can be shared with genomes that did not produce them. This can vary considerably between different viruses. For example: in poliovirus, the capsid proteins are shared, and so act as public goods, but replicase is not^31^; in Influenza, both the capsid proteins and replicase are shared, and so both can act as public goods^32^; in vesicular stomatitis virus, the replicase is shared, but can evolve to become less likely to be shared, so it is a public good, but can evolve to become ‘privatised’^1^.

Viral cooperation can also extend beyond the cell, to include cases where benefits are shared between viral genomes infecting different cells (Fig. 2). For example, animal viruses block the release of interferon from host cells, to suppress the host immune response (Fig. 2)^33^. This suppression is costly to the viral genome encoding the gene for suppression, slowing its replication within a cell, but it provides a public benefit by keeping the local population of host cells susceptible to infection by neighbouring viruses^34^. A number of analogous examples appear to exist elsewhere in viruses, such as when phages encode ‘anti-CRISPR’ proteins that partially overcome bacterial host defences, or when phages communicate via quorum sensing to coordinate their lysis timing^35–37^.

### How to test for cooperation and cheating

We have claimed that viral traits such as producing shared replicase enzymes can be a form of cooperation, which could be cheated by genomes that do not produce these products (Box 2). This requires that the production of these gene products is maintained by natural selection at least partially due to the benefits they provide to other viral genomes. How can this be tested experimentally?

**Fig. 3:**
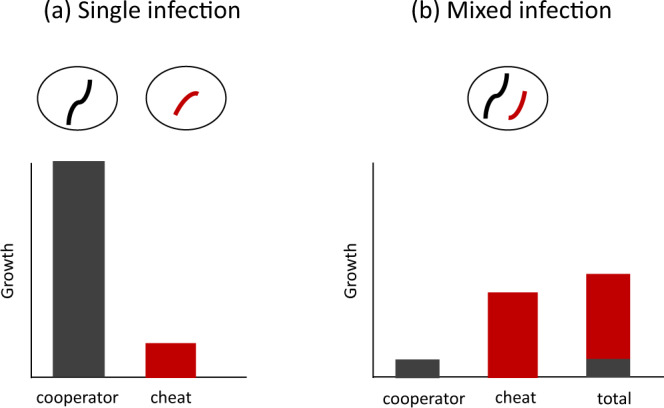
How to test for a cheat. We illustrate the hypothetical results of an experiment in which a cooperator and a cheat are grown **a** separately and **b** together. For two individuals to count as a cheat and cooperator respectively, three conditions must be met: (1) the cooperator must have a higher fitness than the cheat when each are alone; (2) the cheat must have a higher fitness than the cooperator when both are mixed; (3) the cooperator must have a lower fitness in a mixture than it did when it was alone.

In many cases, we can test for both cooperation and cheating with growth assays on strains that do and do not perform the putative cooperative trait, on their own and in a mixed culture^5^. For example, we might have a strain that produces replicase enzymes (putative cooperator), and a strain that does not (putative cheat). If these strains really represent a cooperator and a cheat, we would observe three results:

(i) when grown separately, the cheat would not be able to exploit cooperation, and so would grow slower than the cooperator (Fig. 3);

(ii) when grown together, in a mixture, the cheat would be able to exploit and out-compete the cooperator (Fig. 3);

(iii) the exploitation by the cheat would reduce the fitness of the cooperator, compared to when the cooperator is alone (interference) (Fig. 3).

These experiments are sufficient for demonstrating both cooperation and cheating, provided we are looking at an essential viral trait where only one of the strains has the trait. For example, when one strain encodes a shared replicase enzyme and the other does not. Since the non-replicase strain has been able to grow in the mixed infection (ii), but not in the single infection (i), it must have made use of replicase enzymes produced by the other strain, showing that in this case, replicase is acting as a public good (or in virology terms, it is trans-acting).

In addition, the replicase-producer is out-competed by the non-producer in mixed infection (iii). This shows that the production of replicase enzyme is selected when the benefits of the enzyme go to individuals that do not produce it. The production of replicase enzyme, therefore, fits the evolutionary definition for cooperation here - selection for production depends upon who the benefits go to. Finally, because the non-producer has been replicated, we know that it must have benefitted from the replicase enzymes in the same way that the cooperator does, showing that it is a cheat, and not just a parasite.

In viruses, these three results have been demonstrated clearly in experiments on DI PV1, a cheat of poliovirus that uses capsid proteins produced by cooperative wild-type poliovirus genomes (Box 3). Similar experiments in a wide range of different viruses have determined that replicase, capsid proteins, and other shared gene products, can be cooperative public goods that are commonly exploited by viral cheats that do not produce these gene products (such as defective interfering genomes & satellite viruses)^24,38,39^. ‘Accidental’ experiments have reinforced these findings, by showing that cheats such as defective interfering genomes rapidly spread when viruses are cultured in conditions that favour high coinfection and low relatedness^40^.

These experiments have demonstrated that many essential viral traits that have not previously been thought of as cooperative, such as replicase and capsid proteins, are in fact examples of evolutionary cooperation. At the same time, strains that do not produce these gene products, are cheats. Consequently, this approach highlights the evolutionary similarities between cheats that may differ substantially in their origins or in the molecular details of how they exploit cooperation. This allows us to draw common links between viral cheats and cheats elsewhere in nature, and between viral entities that have previously been studied within their respective subdisciplines^24,39,41,42^.

The experiments shown in Fig. 3 also provide a template for investigating other viral traits that are potentially cooperative, such as so-called ‘antigenic cooperation’, the suppression of CRISPR-based immune systems, or the rate at which phages lyse their hosts^34,43–45^. Furthermore, because this approach requires relatively few mechanistic details to be known, it allows us to place newly discovered or poorly understood viral entities within an existing framework, helping to bring ‘order to the viral universe’^46^.

In some cases, the simple experiments we have outlined will not be sufficient to demonstrate cooperation and cheating, and further experiments will be required. These further experiments will need to unpick the relative costs and benefits of cooperation, the details of which could depend upon the biology of the case being examined examined^34,47–51^. Possible further experiments include examining the consequences of variation in the level of cooperation, or variation in the proportion of the population that are cheats^49,51^. Such experiments can be useful when both strains produce the public good, or if the public good is not essential, and so the putative cheat may gain an advantage over the cooperator for some reason unrelated to cooperation. For example, in a ground-breaking series of studies, Turner and Chao used an experimental approach similar to that outlined in Fig. 3 to show that PhiH2 acts like a partial cheat of Phi6, followed by further work to uncover the mechanisms by which it gains an advantage, confirming that it is a cheat^47,48^.

More broadly, over the last 20 years, experimental methods such as those outlined in Fig. 3 have revolutionised our understanding of cooperation and cheating in bacteria and other microorganisms^14^. Are we at the start of a similar revolution with viruses^26–29^?

Box 3  A  typical  viral  cheat The defective interfering genome ‘DI PV1’ is cheat of poliovirus. DI PV1 contains a large deletion that removes the entire capsid protein region (a)^23,152^. When grown on its own, DI PV1, therefore, produces no viral capsids, and so is unable to spread between host cells (b). However, when wild-type poliovirus and DI PV1 are grown together, copies of DI PV1 can be incorporated into viral capsids produced by the wild-type cooperator. In coinfected cells, the shorter length of DI PV1 means that it is replicated substantially faster than the wild type, and it is also able to enter virions more effectively than the wild type. Consequently, DI PV1 is able to achieve more than 1000 times as many genomes inside viral capsids as the wild-type cooperator, which is a huge fitness advantage (b) (data values from Fig. 1 of Shirogane et al.)^23^.

DI PV1 is a well-known viral entity that has been studied for decades, that also provides a clear fit to the evolutionary definition of a cheat^5,23,31,147,152–155^. It avoids encoding a cooperative trait (producing capsid proteins), but it is able to exploit the cooperation of other genomes (by using capsid proteins they encode). There are direct parallels between the experiments that virologists used to investigate DI PV1, with the experiments that evolutionary biologists typically conduct to examine cheating in bacteria^8^.
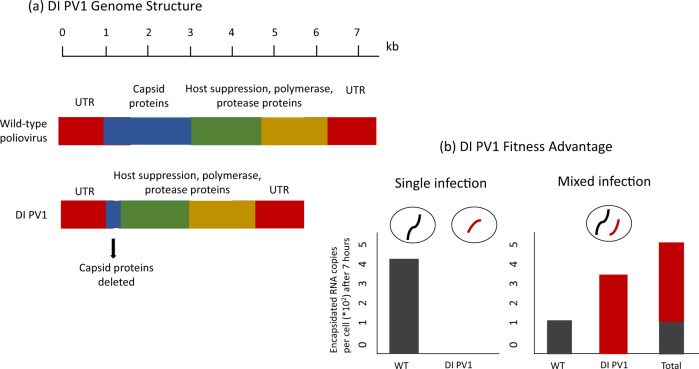

## Where are viral cheats found?

**Fig. 4:**
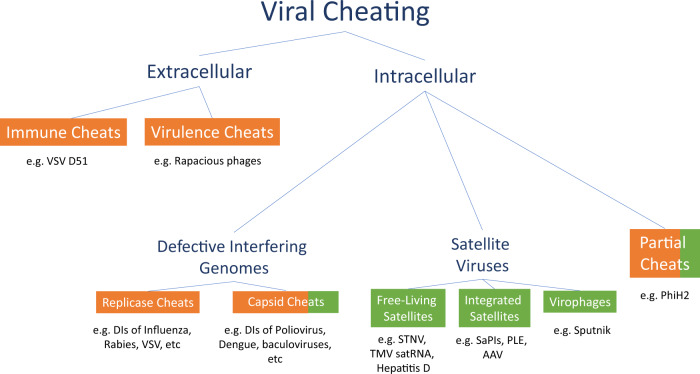
A classification of viral cheats. A variety of different kinds of viral entities are cheats. We first divide viral cheats depending on whether the cooperation that is being exploited is intracellular or extracellular, then according to their origins, and finally, the specific gene products exploited, if known. We also denote the evolutionary timescales at which cheats exist. We divide between short-sighted cheats (in orange), which arise and spread within hosts, but not between hosts, and long-sighted cheats (in green), which spread both within and between hosts, and consequently persist over longer evolutionary timescales. We offer a few illustrative, but not exhaustive, examples of each type of cheat^34,42,44,47,53,62,67–69,96,109,147–150^.

**Fig. 5:**
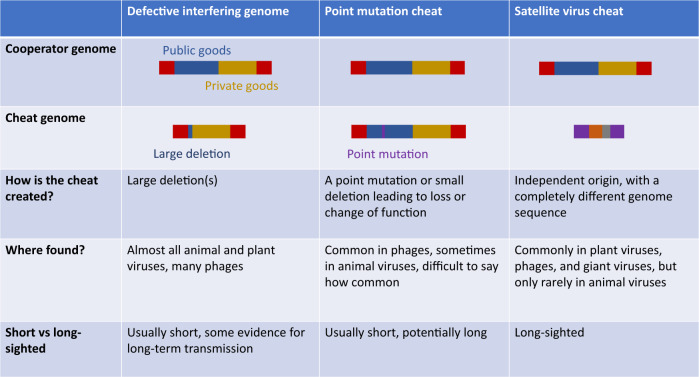
Viral cheat origins. A number of different molecular mechanisms can give rise to viral cheats. The evolutionary perspective highlights the functional similarities between these mechanistically different cheats. Here, we show three different types of cheat, which arise in different ways: large deletion; point mutation; and independent origin.

Cheats are common throughout the viral world. We can divide and classify cheats based on the different kinds of cooperation that they exploit (Fig. 4), and how they originate (Fig. 5). Our first division is between cheats that exploit cooperation between viruses within the same cell (intracellular cooperation) or different cells (extracellular cooperation).

### Intracellular cooperation

(1) Defective interfering genomes, such as DI PV1, are literally defined by the features that make them cheats (Box 2)^38^. Defective interfering genomes emerge spontaneously during infections, via mutations that delete genes for intracellular viral public goods, such as the replicase enzyme, capsid proteins, or proteins that manipulate host cell machinery for the benefit of the infecting viruses^52^. Consequently, they are defective, because they grow less well, if at all, on their own (result i of the three criteria that define a cheat in Fig. 3), and they are interfering genomes because they can increase their own growth rate at the expense of the wild type (results ii & iii from Fig. 3) (Box 3)^38^. Defective interfering genomes are the most prominent kind of viral cheat, having been studied for decades and observed in tissue culture for almost all animal and plant viruses^24,38,52–54^. They can be found in non-segmented, segmented, and multipartite viruses, viruses infecting every major type of host (animal, plant, microbial), and in viruses from across the Baltimore classification (Fig. 4).

Defective interfering genomes are closely analogous to public goods cheats that have been commonly observed in bacteria (Box 2)^7,19,20^. Because they tend to arise through large deletion mutations, defective interfering genomes are usually much shorter than the wild-type virus, which is a key reason why they gain such a large replication advantage in coinfection^15,16,55–59^. In short RNA viruses, defective interfering genomes that lack just one or two cooperative genes can be substantially shorter than the wild type; in larger DNA viruses, defective interfering genomes often lack a large number of genes, suggesting that most of the wild-type genome may consist of genes for cooperative traits^60,61^.

(2) ‘Free-living’ satellite viruses are small viral entities that encode only some, and sometimes none, of the genes required for successful infection, instead relying on gene products encoded by complete ‘helper’ viruses^39,62^. Satellites are varied and can have a range of different effects on their helper viruses, with some satellites being cheats, but others being beneficial for the accumulation of the helper virus, and so appear to be mutualists^39,42,62,63^. In some cases, satellites even encode new genes not found in the helper virus. Cheat satellites share many functional similarities with defective interfering genomes, exploiting similar gene products such as replicase and capsid proteins, and gaining similar advantages through having a shorter length. However, unlike defective interfering genomes, we do not know how most satellites originate, since they tend to share little to no sequence homology with their helper viruses.

Satellite viruses are also much longer-lived than defective interfering genomes, transmitting between hosts and persisting over long evolutionary timescales. In some cases, satellites are themselves exploited by other satellites, or by defective interfering genomes^64^. Satellites are very common in plant viruses, although they can also be found in phages and in animal viruses, including those that infect humans^65,66^.

(3) Some satellite viruses employ a ‘sit-and-wait’ strategy, in which they integrate into host genomes and become dormant, replicating only when a cooperative virus infects their host cell^66,67^. Examples include: adeno-associated viruses, which are present in up to 90% of human genomes; Staphylococcal pathogenicity islands, which are widespread throughout the genomes of gram-positive bacteria, and show evidence of long-term convergent evolution towards cheating; and phage-inducible chromosomal island-like elements (PLEs) in the genomes of *Vibrio cholerae* bacteria, which are involved in coevolutionary arms races that influence when cholera outbreaks occur^42,66,68,69^. This ‘sit-and-wait’ strategy appears to be novel form of cheating, not known in other organisms, and which represents an elegant solution to the problem of a low likelihood of co-transmission with a cooperative virus.

(4) One of the most exciting recent discoveries in virology has been that of giant viruses and their associated satellites, the virophages^70,71^. Giant viruses have very large genomes that can be larger than some bacterial genomes, and construct capsids big enough to be seen with a light microscope^67^. Not long after giant viruses were discovered, virophages were found, which parasitise giant viruses, and appear to be highly abundant^71–73^. They share many similarities with satellite viruses, including a reliance on giant viruses to replicate, origins that are phylogenetically distinct from giant viruses, effects ranging from negative to neutral on giant virus replication, and integration into host genomes^74^. Based on what is currently known about virophages, it seems that most known virophages are cheats, because they exploit the cooperative replication machinery of giant viruses, and potentially also capsids^71^.

(5) Defective interfering genomes and satellites represent extreme forms of cheating, where the cheat has completely lost the ability to replicate on its own, and is entirely dependent on the cooperator. Less extreme forms of cheating are also possible in viruses, where the cheat does better when it exploits the cooperator, but can still survive and replicate on its own. For example, we have already discussed how PhiH2 is a cheat of the phage Phi6, out-competing Phi6 in mixed infections and losing in single infections. However, PhiH2 is only a partial cheat, because it still retains some ability to replicate itself in the absence of Phi6^47^. This is analogous to many kinds of facultative cheating elsewhere in nature, such as bacteria that only partially downregulate the production of a public good^21,50^. Partial cheating may be rarer in viruses than in other organisms, perhaps because viruses’ smaller genomes lend themselves more easily to simpler ‘all-or-nothing’ mutations that completely knock out a function, resulting in complete cheating^60^.

### Inter-cellular

Cooperation and cheating also occur between viral genomes infecting different cells.

(1) In animals, cells that are infected with a virus often produce interferons, a group of signalling molecules that spread to nearby cells and trigger antiviral defences^33^. Many viruses produce a molecule to block the release of interferon. The production of this interferon-blocking molecule is costly, but it also provides a benefit to viruses in nearby cells, by keeping the local population of host cells susceptible to infection^34^. Consequently, interferon-blocking represents a form of costly cooperation between viruses in different host cells^75^.

In Vesicular Stomatitis Virus (VSV), D51 is a cheat mutant that exploits cooperative interferon blocking. D51 avoids the cost of blocking interferon, consequently replicating more quickly in infected cells, and spreads at the expense of wild-type VSV when both are grown together^34,75^. However, when D51 is grown on its own, no interferon-blocking proteins are produced, and so it quickly becomes extinct because local host cells activate their antiviral defences. Viral mutants that are less effective at suppressing interferon are common in natural infections of many different viruses, including important human pathogens such as SARS-CoV-2 and Influenza A, potentially suggesting that this is a widespread form of viral cheating^76,77^.

### Where else could viral cheats be found?

We expect that the cooperative viral traits we have described are only a fraction of those that exist (Fig. 4)^78–81^. As we explore more of the viral universe, we expect to find new kinds of cooperative traits, and these may be exploited by new kinds of viral cheat. Some types of trait that could be cooperative but where cheats have not yet been found include the production of anti-CRISPR proteins by phages of *Pseudomonas* bacteria^43^; the production of arbitrium quorum-sensing molecules in phages of *Bacillus* bacteria^37^; and the production of depolymerase enzymes, that many phages produce to break down bacterial cell walls^82^.

A broad across-species understanding of cheating in viruses is currently held back by technical limitations and taxonomic bias. For example, we have relatively few examples of ‘point mutation’ cheats compared to defective interfering genomes, but is this because they are rarer, or just because they are harder to detect? Satellite viruses are found commonly in plants and phages, but less commonly in animal viruses; meanwhile, defective interfering genomes are found commonly in animal and plant viruses, but less commonly in phages. Are these real patterns, or just statistical artefacts stemming from the fact that we have only studied a small and biased subset of viruses in depth? Technological advances that are allowing unbiased metagenomic sequencing across a broad range of viruses, coupled with sequencing technology that can reveal within-host viral variation, could help to solve these limitations.

## When are cheats favoured?

The same factors that govern the spread of cheats elsewhere in nature can be used to understand when viral cheats will spread. Once a cheat has arisen, viral or otherwise, the rate at which it spreads will be determined by the opportunities that are available for cheats to exploit cooperators. These opportunities are predicted by the genetic relatedness between interacting individuals (Box 4). When relatedness is high, cooperators mostly interact with other cooperators, and cheats with other cheats; this makes it difficult for cheats to spread. In contrast, when relatedness is low, there can be mixing between cooperators and cheats, making it easier for cheats to spread.

The spatial scale at which relatedness predicts the spread of a cheat within a host will depend on the spatial scale of the cooperative trait in question. For example, for a viral public good that is shared within a cell, as is often the case with replicase or capsid proteins, it is the relatedness between the viral genomes infecting the same cell that matters. For this kind of trait, cheating is favoured when cheats commonly coinfect cells with cooperators (low relatedness within cells). In contrast, for a public good that is shared between cells, such as interferon-suppression, it is the relatedness among the nearby group of infected cells that matters (low relatedness among cells, but either low or high relatedness within cells).

Considering the spread of cheats among hosts, if only a small number of viral genomes are transmitted when new hosts are infected, then relatedness would be high when transmitting between hosts. Consequently, cheats would be less likely to spread successfully to new hosts, since they would find themselves surrounded by other cheats, without cooperators to exploit. In contrast, if large numbers of viral genomes are transmitted, then relatedness could be low when infecting new hosts, allowing cheats to spread between hosts, as well as within them.

Relatedness can be calculated in the same way across many different biological systems, facilitating broad comparisons. Research has shown that a high relatedness between interacting individuals plays a clear and consistent role in favouring cooperation, at all levels of biology^14^. Recent work on viruses has suggested analogous patterns, where a high relatedness favours cooperation, and a lower relatedness can favour cheating [REFS]. Other factors are also important in determining whether cooperators or cheats prosper, such as the costs and benefits of cooperation^34^. However, the costs and benefits of cooperation will often depend on biological details that are specific to each system, and so it is more difficult to draw universal predictions.

Box 4  Genetic  relatedness Genetic relatedness is a statistical concept, describing the degree of genetic similarity between social partners, over and above genetic similarity to the average individual in the population^146^. In the simplest case, if *N* genetically distinct and equally abundant viral genome copies infect a cell, then average relatedness within the cell will be *r* = 1/*N*. This comes from the average of individuals being related by *r* = 1 to their clonemates, and by *r* = 0 to individuals in the other *N* − 1 lineages.

Because relatedness is a measure of genetic similarity, it can be defined for any locus in the genome^146^. For determining when cooperation will be favoured, the relevant locus is the gene that controls the cooperative trait. This could be a single allele, or it could be a whole section of the genome (Fig. 5). If the gene for a cooperative trait has been lost, then the relatedness for that trait, between individuals that have the gene, and individuals that do not, will be *r* = 0.

Relatedness changes the benefit of investing into public goods (Box 2). When relatedness is high, interacting individuals are more likely to share genes. When a cooperator interacts with relatives, the benefits of cooperation are likely to be returned to other individuals who also carry the gene for cooperation. Consequently, cooperation provides an indirect (kin-selected) benefit, and will be favoured. In contrast, when a cheat interacts with relatives, these are likely to also be cheats, and so they will unable to exploit cooperation. Consequently, when relatedness is high, cheats will not spread.

In contrast, when relatedness is low, interacting individuals are less likely to share genes, and so cooperation provides a smaller indirect (kin-selected) benefit. At the same time, low relatedness means that cheats are more likely to interact with cooperators, meaning they will be able to exploit them, and hence spread.

Empirically, relatedness has been shown to favour cooperation at all levels of biology, from animals such as humans, birds and bees, all the way across to bacteria, viruses, and simple RNA replicators^14^.

### Determinants of viral relatedness

Relatedness will be influenced both by viral biology and by the physical features of the viral environment. Viral traits such as superinfection exclusion will increase relatedness within cells, by restricting infection to just a small number of genomes^83^. In contrast, traits such as collective transmission can have the opposite effect, by allowing multiple viral genomes to infect the same host cells, especially when they bring together genomes that have come from different cells^84,85^. Other traits that can determine relatedness are influenced by a combination of viral biology and physical features of the environment. For example, relatedness will be lower when mutation rates are higher, or when virions disperse further, since these factors increase the likelihood that co-infecting viruses are genetically distinct^86^.

### Viral relatedness in artificial infections

The role of relatedness has been demonstrated unintentionally numerous times, when growing viruses. Artificial environments, such as tissue cultures or bioreactors, often involve large numbers of viruses compared to the number of host cells, and can be well mixed, with weak spatial structuring. This means that viruses often achieve very high rates of coinfection in artificial environments, with potentially hundreds to thousands of viruses infecting each host cell, and where those viruses are likely to have come from different host cells. In these environments, large numbers of viruses are typically used to seed each new culture, creating persistently low-relatedness conditions that should favour cheats spreading.

Consistent with this, viral cheats such as defective interfering genomes are extremely common in viral tissue culture infections, and have long been an issue in industrial processes that depend on culturing viruses, such as the production of vaccines, biopesticides, or vectors for gene therapy^40,87,88^. Many techniques that industrial producers use to increase yields, such as periodic bottlenecking, are effective because they increase relatedness, making it harder for these cheats to spread^89^. The close parallels between these methods and the formal tests for cooperation and cheating highlight the importance of cooperation and cheating in viral population dynamics (Fig. 3).

### Viral relatedness in natural infections

The extent to which cheats can spread within and between natural hosts will depend on relatedness in natural viral infections. We can use a number of avenues to estimate this. A number of studies in animal viruses, plant viruses, and phages, have found that cells are often infected by multiple viral genomes, suggesting that relatedness can be relatively low within infected cells. For example, in Guinea pigs infected with influenza A^90^, and turnip plants infected by cauliflower mosaic virus^91^, 5-15 and 2-13 viral genomes infected each host cell, respectively; in marine Gammaproteobacteria, half of the infected bacterial cells contained multiple actively replicating phage species^92^. In viruses such as Influenza and HIV, reassortment and recombination between genetically different viruses are relatively common, further suggesting that relatedness can be relatively low within infected cells^93,94^.

There is also evidence that relatedness can be relatively low within host tissues. For example, viruses modified to be entirely dependent on coinfection can grow robustly in animal hosts, and defective viral genomes are often maintained within natural hosts^94–99^. These findings suggest that relatedness remains low over multiple rounds of cellular infection within natural hosts. Defective viral genomes also accumulate at different rates within different host tissues, suggesting that relatedness may vary across different host tissues^100^.

Relatedness between hosts will largely depend on the number of viral genomes that are transmitted to each new host, termed the bottleneck size. In animal viruses, bottlenecks are often narrow, resulting in few viral genomes and therefore high relatedness. For example, in animal viruses such as HIV, SARS-CoV-2, and Hepatitis C, infections tend to be initiated by just a single viral genome^101–105^. In plant viruses, bottlenecks are often wider, resulting in more viral genomes being transmitted, and potentially lower levels of relatedness, such as in Cauliflower Mosaic Virus, where infections can be initiated by 1-13 genomes^91,104^. Bottlenecks can also vary within the same virus, depending on the transmission route. For example, in Influenza A, bottlenecks are larger when transmission is via direct contact, than when it is via aerosol^106^. Some of the widest between-host bottlenecks are found when viruses transmit between hosts using collective infectious units, which can sometimes allow hundreds of viral genomes to infect a new host^107,108^.

Together, these results suggest that relatedness can often be relatively low within cells and within tissues, implying that viral cheats should often be able to spread within natural hosts. However, these cheats will only persist over epidemiological timescales when between-host bottlenecks are wide enough to allow relatively low relatedness in each newly infected host. If between-host bottlenecks are small, then cheats will infect new hosts without cooperators and be unable to spread. These patterns are consistent with the fact that animal viruses often have cheats such as defective interfering genomes, which persist within hosts, but only rarely spread between hosts^24^. In contrast, cheats that spread both within and between hosts, such as satellite viruses, seem to be more common in viruses that have wider transmission bottlenecks; these longer-lasting cheats are found more commonly in plant viruses, and viruses that transmit between hosts inside collective infectious units, such as baculoviruses^62,109^.

### Why don’t cheats take over?

Given the potential benefits of cheating, what stops viral cheats from spreading to fixation after they have arisen and started to spread? Will cheats inevitably win, or can cooperators and cheats coexist?

Even when cheats are able to spread initially, they can be prevented from spreading to fixation by frequency dependence. A common feature of cheating is that the relative fitness of cheats decreases as they become more common—termed negative frequency dependence^49^. Because cheats spread by exploiting cooperators, they experience the greatest fitness advantages when rare, when most other individuals they interact with are cooperators. In contrast, as cheats become more common, they interact with other cheats more frequently than with cooperators, and so their fitness advantage decreases. Consequently, cheating can be self-limiting, and even cheats that have substantial fitness advantages when rare may end up coexisting with cooperators rather than driving cooperators extinct.

Another possibility is that cooperators can adapt to the presence of cheats, in a way that limits their spread^110–112^. In vesicular stomatitis virus (VSV), wild-type cooperators can evolve a form of resistance to cheats, by changing the recognition sequence for the replicase enzyme, so that it still replicates the wild-type cooperator, but no longer replicates the defective interfering cheat genome^113^. Alternatively, viruses could evolve to manipulate population structure in ways that increase relatedness, preventing cheats from spreading (Box 4). For example, viruses could decrease the number of viral genomes that collectively transmit to new cells (smaller collective infectious units)^84,85^, or exclude additional viral genomes from infecting the same host cell (superinfection exclusion)^83,114,115^.

## Why should evolutionary biologists care?

### Viruses versus other lifeforms

A comparison of cheating in viruses versus other organisms raises the question of whether cheating in viruses is the same as cheating elsewhere in the living world (Fig. 1)? We argue that while it is clearly analogous, viral biology leads to important differences. These include:

(1) The high mutation rate and simple genome of viruses means that mutations to cheating can happen relatively easily^116^. For example, defective interfering genomes regularly emerge de novo in viral infections^99^. This high mutation rate allows cheats to frequently arise and spread, even when they would not be maintained long-term.

(2) Viruses can benefit from cheating in a unique way. In addition to the benefit of avoiding cooperation, viral cheats can also gain an appreciable benefit through losing or otherwise modifying those now-redundant cooperative genes^22,58,117^. Cheat genomes can therefore be replicated and encapsidated much faster than cooperative viruses, giving an additional advantage with no clear analogue elsewhere in the natural world.

(3) Consequently, the short-term advantages of cheating in viruses can be exceptionally high. Viral cheats can achieve a 1,000-fold or higher replicative advantage over cooperators, which is orders of magnitude higher than the fitness advantages seen in cuckoos, non-producing bacteria, or other cheats^8,21,23^.

(4) However, this fitness advantage of cheats is often transient at a local scale. For example, many viral cheats emerge easily, and spread rapidly, within a host, but then show poor or even non-existent transmission to new hosts^85^.

Taken together, these features mean that cheating can be both common and transient in many viruses. Viral cheats are therefore special in the extent to which they are often characterised by ‘boom and bust’ dynamics. More so than in other organisms, viruses could be selected to evolve mechanisms to avoid generating cheats, and/or reduce exploitation by cheats.

Not all viral cheats are transient. We can place viral cheats on a continuum between ‘short-sighted’ and ‘long-sighted’ cheats^118^. Defective interfering genomes are short-sighted cheats that arise and spread transiently, mostly within but not between hosts, with boom and bust dynamics. Satellite viruses are long-sighted cheats that spread both within and between hosts, allowing persistence over long evolutionary timescales. Long-sighted cheats are therefore more similar to some forms of cheating observed in animals, such as cuckoos, which persist over long timescales, exploit phylogenetically unrelated cooperators, and coevolve with cooperators over long timescales, often resulting in complex adaptations. In contrast, short-sighted cheats may be closer to public goods cheats in bacteria, or cancer in Eukaryotes, since they are generated de novo each generation, are phylogenetically descended from cooperators, and coevolve with cooperators over much shorter timescales^5,21,110^.

### Viruses make model cheats

The unique features of viral cheating make viruses excellent model organisms for studying cheating. Cheats may be both more common and easier to find in viruses than in other organisms. The relatively small genomes and short generation times of viruses mean that it is often easy to link genotype with phenotype, allowing us to identify cheats relatively easily, and to follow evolutionary dynamics over time^119^. The large amounts of clinical and environmental genomic data allow the ecological and coevolutionary dynamics of cheating to be studied in nature^12,97^. These studies can then be complemented with manipulative laboratory experiments that are more feasible in viruses than in other organisms^119^.

### Novel evolutionary problems

Cheating in viruses raises novel evolutionary problems. In the laboratory, viruses can be genetically modified so that it is harder for cheats to arise through mutation^120^. Have viral genomes naturally evolved similar strategies to limit the emergence of cheats, for instance by linking cooperative genes with essential private functions that cannot be cheated^121^? There are a number of instances where it looks like this might have happened: in measles virus, the C protein inhibits the formation of defective interfering genomes^122^; in polioviruses, defective genomes that lack sections of the replicase gene are unable to be incorporated into virions, and so ‘replicase-cheats’ do not evolve (although ‘capsid cheats’ do)^31^; in Flock House Virus, successful cheats contain two large deletions in the genome, because cheats with just one large deletion lose essential functions in the middle of the genome that cannot be complemented by coinfecting with another genome^123^.

## Why should virologists care?

### Adaptation in viruses

An understanding of cheat-cooperator dynamics can inform how we think about viral populations. Viral cheats appear to be very common, but also have strongly negative consequences for viral infections, and for other viral variants. This challenges the idea that viruses should be defined at the group or ‘quasispecies’ level, because it suggests that the potential for conflict is likely to prevent adaptations that are solely for the benefit of the group of viruses^124,125^. Therefore, we should not necessarily expect viral populations to evolve as coherent groups, nor to be adapted towards any collective goal.

### New predictions: molecular and genomic

Social evolution theory makes broad predictions about the genomic and molecular biology of viral cheats, but in many cases, more specific predictions will depend on linking more detailed viral biology with the evolutionary biology of cheating.

For instance, we can predict that intracellular viral cheats are likely to exploit trans-acting gene products such as replicase enzymes and capsid proteins, since these can be public goods (Box 2). Molecular details could then allow more precise predictions to be developed, such as why viral cheats sometimes exploit capsid proteins, sometimes replicase, and sometimes both.

We can also predict which viruses are most likely to be affected by cheats. Viruses that encode many trans-acting (social) gene products should be more likely to be exploited by viral cheats, as should viruses that experience higher rates of coinfection, such as those with mechanisms for collective infection. Pleiotropy will make it harder for viral cheats to emerge when it links trans-acting (social) with cis-acting (non-social) functions, such as in the replicase gene of poliovirus^31^. However, pleiotropy may make it easier for viral cheats to emerge when it links multiple social gene products, as occurs in the phage M2, since this allows a single mutation to exploit multiple forms of cooperation^22^.

We might expect viral cheats to be more common in short RNA viruses, where mutations rates are high and a single cooperative gene can comprise a large fraction of the viral genome^116^. On the other hand, cheating could be easier in large DNA viruses, which are less likely to have pleiotropy linking social with non-social traits, and may contain a larger number of cooperative genes, allowing cheats to lose many genes and become much shorter than cooperators^61^. Formal tests of these kinds of evolutionary prediction will become possible when we have more unbiased sampling of cheats across different types of virus, and more thoroughly annotated viral genomes.

We would also expect to see long-term genomic consequences of cheating. A number of resistance mechanisms are possible in response to viral cheating, such as pleiotropy that links social (trans-acting) to non-social (cis-acting) genes, modifying trans-acting genes to be more cis-acting, or decreasing the size of collective infectious units^113,120^. Different types of virus may vary in the extent to which resistance is possible. For instance, it may be less feasible for viruses with segmented genomes to evolve replicase enzymes that are more cis-acting, because this would require simultaneous mutations on several genome segments at once. Resistance mechanisms may also complicate evolutionary predictions, because they are most likely to evolve in viruses that experience lots of cheating, but they are then likely to decrease the prevalence of cheating in those viruses, potentially creating a ‘chicken-and-egg’ problem.

More broadly, the potential for coevolution between cooperators and cheats could complicate many of the predictions that we have laid out in this section. For example, viruses that have greater numbers of cooperative genes may be more resistant to cheating, and so we may expect to find fewer cheats, not more. Teasing apart these coevolutionary complications can be difficult, but this kind of complication is not unique to viruses, and comparative approaches that start with simple predictions based on evolutionary theory have been empirically successful in other areas of social evolution^14^.

### Explaining clinical outcomes

Understanding cheating could help explain why the same virus can lead to different clinical outcomes in different patients. For example, if cheats spread during an infection, then this could lead to a lower viral load and lower virulence. Consistent with this, infections with both influenza and ebola viruses that contain a larger number of defective interfering genomes are less likely to lead to severe clinical outcomes, such as admittance to the intensive care unit^126,127^. However, one potential complication here is that higher viral loads can allow cheats to better exploit cooperators, in which case cheats might be more likely to be found in infections with higher viral loads, and more severe clinical outcomes.

Social dynamics could help to explain why variants that arise during individual clinical infections often fail to spread between hosts to become dominant on an epidemiological scale^128,129^. One possibility is that some of these variants could be short-sighted cheats, which can spread within a host, but which are unlikely to co-transmit with cooperative viruses between hosts, due to small between-host bottlenecks^103,104^.

### New treatments

Cheating can be exploited as a mechanism to disrupt viral infections, and social evolution could inform how we approach this. Therapeutic interfering particles (TIPs) are synthetic viruses designed to exploit wild-type virus cooperation, and to suppress viral infections by acting as a cheat, mimicking defective interfering genomes^130,131^. They are analogous to other types of ‘cheat therapy’ being developed against bacteria^132,133^. Animal challenge studies suggest that therapeutic interfering particles can be highly effective, both as prophylactic and as a treatment post-infection, against viruses including Lassa virus, Chikungunya virus, Influenza A virus, and SARS-CoV-2^100,134–139^.

One advantage of therapeutic interfering particles is that they exploit predictable features that are common to all viruses, and so it could be relatively quick and straightforward to develop therapeutic interfering particles against a novel virus. Another advantage is that some therapeutic interfering particles are effective even against viruses that are relatively distantly related to the virus that they came from—a feature they share with many satellite viruses^131^. For example, defective interfering genomes of the Chikungunya virus can be effective against other alphaviruses, such as Sindbis virus and O’nyong-nyong virus^100,138^. Consequently, it could be relatively quick to deploy therapeutic interfering particles against a novel virus, either by designing new ones or adapting existing ones.

Social evolution theory could help to design effective therapeutic interfering particles (TIPs), because it offers tools for answering analogous questions to those being posed in therapeutic interfering particle research. For example, compare ‘when do TIPs suppress wild-type viruses?’, ‘can TIPs be maintained in the population?’, and ‘can TIPs revert to being fully infectious viruses?’, with ‘when do cheats win?’, ‘when does frequency dependent selection maintain cheats and cooperators at equilibrium?’, and ‘can cooperation be regained?’.

A social evolution perspective could also help us determine how to use therapeutic interfering particles effectively, by focusing on the evolutionary dynamics of natural viral cheats. Before using a synthetic cheat to control a viral infection, we would first want to know what kinds of cheat affect the virus naturally, and how the virus responds to them. Are some viruses more susceptible than others to being exploited by cheats? Which viral cheats are able to spread between hosts, and why? In what ways do viruses evolve resistance to cheats, and can cheats coevolve in response? These are all pressing questions about the natural history of viral cheats, which also have clear applications in informing how therapeutic interfering particles could be used safely and effectively.

## Where next?

The study of viral cheats offers amazing opportunities to both evolutionary biology and virology. However, the biggest obstacle in this field is that most of the existing empirical work has been done in the laboratory, either in tissue culture or in model hosts. To move forward, we need to understand the role that cheats play in the epidemiological and evolutionary dynamics of viruses in their natural environment, which includes humans, crops, and livestock. Fortunately, exactly the right kind of data is now being collected, as next-generation sequencing technology is increasingly being used to monitor viral outbreaks and to chart the enormous unexplored diversity of viruses^79–81,140–142^. The next steps will involve harnessing this rapidly advancing technology in order to test and expand evolutionary theory about viral cheats.
